# How to Stop Disagreeing and Start Cooperatingin the Presence of Asymmetric Packet Loss

**DOI:** 10.3390/s18041287

**Published:** 2018-04-22

**Authors:** Oscar Morales-Ponce, Elad M. Schiller, Paolo Falcone

**Affiliations:** 1 Department of Computer Engineering and Computer Science, California State University Long Beach, Long Beach, CA, 90840, USA; oscar.moralesponce@csulb.edu; 2 Department of Computer Science and Engineering, Chalmers University of Technology, Göteborg, Sweden; 3 Department of Electrical Engineering, Chalmers University of Technology, Göteborg, Sweden; falcone@chalmers.se

**Keywords:** dependable communication protocols, cooperative systems, information quality in intelligent transportation systems

## Abstract

We consider the design of a disagreement correction protocol in multi-vehicle systems. Vehicles broadcast in real-time vital information such as position, direction, speed, acceleration, intention, etc. This information is then used to identify the risks and adapt their trajectory to maintain the highest performance without compromising the safety. To minimize the risk due to the use of inconsistent information, all cooperating vehicles must agree whether to use the exchanged information to operate in a cooperative mode or use the only local information to operate in an autonomous mode. However, since wireless communications are prone to failures, it is impossible to deterministically reach an agreement. Therefore, any protocol will exhibit necessary disagreement periods. In this paper, we investigate whether vehicles can still cooperate despite communication failures even in the scenario where communication is suddenly not available. We present a deterministic protocol that allows all participants to either operate a cooperative mode when vehicles can exchange all the information in a timely manner or operate in autonomous mode when messages are lost. We show formally that the disagreement time is bounded by the time that the communication channel requires to deliver messages and validate our protocol using NS-3 simulations. We explain how the proposed solution can be used in vehicular platooning to attain high performance and still guarantee high safety standards despite communication failures.

## 1. Introduction

Vehicular ad hoc networks (VANETs), just like any other wireless mobile network, are prone to packet loss in ways that are hard to predict due to the unregulated motion of the transmitting nodes and other objects in their environment as well as the complex patterns of radio way propagation. There is a need to ensure that the collected sensor data have enough quality for the intended purposes and that the vehicular system can monitor its input so that the system control can be made predictable. We propose a dependable communication protocol that improves the use of data according to their quality in manners that meet the performance needs, deployment scale, mobility, and criticality of the applications.

Recently, the US Department of Transportation (US DoT) has announced that all new vehicles will be equipped with vehicle-to-vehicle (V2V) communication and be able to talk with each other [1]. Connected vehicles open a great variety of applications that will improve the mobility on the roads, reduce the greenhouse gas emissions and maintain high safety standards on the roads. Vehicles broadcast their own sensory information such as localization, direction, speed, acceleration, intention, etc., and receive this information from nearby vehicles. Currently, dedicated short range communication (DSRC) devices allow broadcasting up to 10 times per second. This information is used to identify the risk in real-time and adapt the trajectory accordingly. However, unilaterally changing the trajectory can propagate the risk to nearby vehicles, creating a cascade effect. To minimize this phenomenon, all nearby vehicles must be aware of the intention of each other nearby vehicle. In other words, all nearby vehicles must agree on their trajectory to be used.

Let us consider two trajectory modes: (1) Cooperative mode where the trajectory attains high performance by considering all the sensory information of every nearby vehicle, i.e., all nearby vehicles have common knowledge. Therefore, the cascade effects will depend mainly on the time that the information is disseminated. The cooperative mode is suitable for time-critical applications that attain optimal performance, such as platooning, unmanaged intersection crossing with minimum waiting time, etc. Common knowledge requires that every vehicle knows that each other cooperating vehicle knows the intention of the others. In other words, all vehicles must agree on the trajectory mode to be used. (2) The autonomous mode where each vehicle computes its trajectory based only on its on-board sensors. Therefore, the autonomous mode is suitable when vehicles cannot correctly communicate. Although autonomous trajectories have lower performance than the cooperative trajectories, they are safe even if no communication is available.

To operate in cooperative mode, all nearby vehicles must be aware that everyone else is in cooperative mode, i.e., they must have reached an agreement. In a normal operation all the vehicles receive the message in a timely manner and, consequently, they are able to reach an agreement. However, wireless communications are prone to failure due to physical phenomenons such as interference, scattering, multipath fading, etc. Hence, it is impossible to deterministically reach an agreement [2] (Theorem 5.1). Therefore, any protocol will necessarily exhibit disagreement periods. We observe that vehicles can still safely cooperate to attain the highest performance even when they experience communication failures provided that these occur for sufficiently short periods. However, when vehicles experience long periods of communication failures, the inaccuracy of the information such as position, speed, intention, etc. increases. Therefore, the safety hazard also increases. Then, the natural question is whether there exists a protocol that guarantees that the disagreement period is bounded by a constant time regardless of the period of communication failures.

In this paper, we address the following research question: How can cooperative systems be used to attain the highest performance without compromising the safety in the presence of communication failures? In our approach, vehicles operate in cooperative mode with the highest level of performance when they are able to agree on the last used trajectory. Otherwise, vehicles switch to the autonomous mode. We present a communication protocol that guarantees that the disagreement periods occur only for one communication round in the presence of communication failures, which is the minimum possible since any protocol exhibits disagreement periods.

### 1.1. Related Work

In the context of distributed algorithms, the term consensus appears in the literature when referring to different problem definitions. Distributed uniform consensus considers the selection of exactly one value from a set of values proposed by members of, say, a vehicular system. A common definition for the exact (uniform) consensus problem requires satisfying the following three properties.

- Agreement: the members always agree on the selected value.
- Validity: the selected value was indeed proposed by a member.
- Liveness: eventually all non-failing members decide on a single value.

We note that this is not the only kind of uniform consensus that appears in the literature [2,3,4].

The term approximate consensus [5] refers to a collection of approaches for calculating a function in a distributed and approximated manner. For example, in the average consensus problem [6], every member proposes a number and the members need to calculate the average value of these proposals. The problem motivation also assumes that no member should collect these values and calculate the average directly, i.e., in a centralized manner. Instead, pair-wise interactions let the members calculate a number that is not too far from the average. In each interaction, a pair of nodes adjusts their proposals. For example, the proposal that is higher (among the two that interact) is reduced by ϵ and the lower one is raised by ϵ, where ϵ is some small predefined constant. This process continues until the difference between the proposals becomes smaller than the approximation threshold and the members can decide.

#### 1.1.1. Exact (Uniform) Consensus

The solution of uniform consensus is required to terminate within a bounded time. Termination is achieved once all members have decided on a common value. This exact version of consensus has a clear advantage over approximate consensus approaches [5] due to the simplicity of the system design from the application programmer perspective. The exact consensus approach, in contrast to the approximate one, rests on a foundation of clearly defined requirements and is amenable to formal methods and analytical validation.

A number of impossibility results consider distributed consensus in general (see [7,8,9]). In [2], the author shows that the presence of communication failures makes it impossible to deterministically reach uniform consensus (Theorem 5.1) and any r-round algorithm has a probability of disagreement of at least $\frac{1}{r+1}$ (Theorem 5.5). This implies that there are no (deterministic) guarantees that vehicles can reach consensus within a bounded time in the presence of communications failures. Moreover, when the communication failures are too frequent and severe, the members might fail to reach consensus for an unbounded number of consecutive times. We therefore relax the agreement requirement since we prefer to focus on solutions that offer early fallback strategies against the risk of having disagreements for more than a constant number of rounds.

The existing literature on uniform consensus algorithms with real-time requirements often does consider failing members. However, it often assumes the availability of timed and reliable communication channels. For example, [10] proposes an algorithm that reaches agreement within a period that is sublinear in the number of members and maximum message delay. In [11], the authors provide a time-optimal consensus algorithm that reaches consensus in time O(D(f+1)) in the worst case where *D* is the maximum message delay and *f* the maximum number of members that stop (fail) to compute. RTCAST [12] is a real-time group communication protocol that assumes that availability of a network with a topology of a ring, such that any two neighbors on the ring communicate via a real-time communication service that provides QoS guarantees, maintains a connection, overload protection, and fairness. We consider plain ad-hoc communications in single hop networks that have no QoS guarantees. The real-time local area network (RTLAN) architecture [13] permits applications to dynamically specify their communication timing requirements and provides mechanisms to guarantee these requirements. The authors consider several approaches, such as priority assignment, real-time virtual circuit, and reservation. This work considers simple ad-hoc communications (in a peer to peer mode) that do not provide the above opportunities.

#### 1.1.2. Approximated Consensus

Approximated consensus estimates the value of a specified function that its input is given in a distributed manner; each member provides a values proposal. For example, the problem of *average consensus* requires members to reach an approximated agreement to the average of their initial values within a bounded number of communication rounds. Dominguez-Garcia and Hadjicostis consider an application for average consensus in the area of energy control of resources. The applications of average consensus also include vehicular systems with recent examples in vehicular coordination, target tracking in wireless networks and interconnected traffic lights controller.

The mobile nodes in VANETs can move rapidly. Packet loss can occur due to signal interference and rapid mobility. Kempe, Dobra, and Gehrke study the connection between distributed gossip and average consensus. They show how to use uniform gossip for computing sums, averages, random samples, quantiles to name a few functions. For their settings (that consider synchrony and no mobility), they show convergence within exponential time. Bènèzit et al. consider weighted-gossip for dealing with directed networks (that are strongly connected). The literature on weighted consensus also includes recent studies on optimal weighting matrix for general network topologies as well as closed-form expressions for the convergence time of algorithms on cycles, paths and grids. Weighted consensus has a number of important applications, for example, as a basis for object tracking.

Fagnani and Zampieri studied average consensus with a fault model that included link failures. Kar and Moura studied the same problem and similar model in the area of wireless sensor networks (WSNs). More recently, Su and Vaidya considered Byzantine behavior under the assumption that the communication graph does not change. The literature about average consensus also includes system settings that model mobility via topology changes and communication delays (as long as the communication delays are bounded by a constant and the network is strongly connected in some sense, i.e., no permanent joins and leaves). For example, Hadjicostis and Charalambous show that their version of average consensus converges in the presence of dynamic topology as long as the communication delays are bounded. Moreover, when the communication delays are minimal, i.e., all packets arrive within a single communication round, convergence occurs with 5 to 10 rounds for the case of fully connected graphs (and the number of rounds increases as the delays become longer). When there are no communication delays, our algorithm reaches agreement within one or two communication rounds (for the case of fully connected graphs and within the order of the diameter of the communication graph for the general case). Moreover, our algorithm recovers within a minimal number of communication rounds after any finite period of faulty communication.

#### 1.1.3. Membership Services

Group communication systems [14] treat a group of participants as a single communication endpoint. The group membership service [15,16,17] monitors the set of recently live and connected participating system components whereas the multicast service delivers messages to that group under some delivery guarantees, such as delivery acknowledgment. In this paper, we assume the availability of a membership service and a best-effort (single round solution) dissemination (multicast) protocol that has no delivery acknowledgment (but might offer probabilistic guarantees). This combination of services allows the system to ban failing node using the membership service, and to overcome transient packet losses using the gossip guarantees.

We clarify that, broadly speaking, membership services sometimes require reaching a uniform agreement via the execution of a uniform consensus protocol. For example, virtual synchrony does not require the execution of uniform consensus for delivering multicast messages in total order when there are no membership changes. In addition, the membership service does not require reaching uniform consensus in the presence of membership changes as long as the group leader stays up and connected. However, when the group leader fails, the membership service uses a procedure for achieving uniform consensus. See for details.

#### 1.1.4. Applications to the Proposed Protocol

The application set of the proposed protocol includes distributed tasks that require: (i) repeated executions of uniform consensus and (ii) convergence within one communication round, but can (iii) tolerate disagreement (due to communication failures) for a minimal period. For example, we consider applications in which the individual vehicles estimate their ability to cooperate according to the sensory information quality and communicate their maximum supported cooperative operation mode according to [18,19]. The architectural component presented in [18,19] is called a *safety kernel* and it allows the vehicular system to use its sensory data to decide on its current level of service.

The algorithm presented here is the one that allowed the safety kernel of [18,19] to consider the data quality of all vehicles when taking this decision. In an earlier version of this work [20], we proposed the studied solution along with experiments for validating it. Note that this paper adds the formal correctness proof of that solution, which does not appear in [20].

### 1.2. Our Contribution

We study an elegant solution for cooperative vehicular systems that have to deal with communication uncertainties. We base the solution on a communication protocol that, we believe, can be well understood by designers of safety-critical, automated and cyber-physical systems. We explain how the designers of fault-tolerant cooperative applications can use this solution to deal with communication failures when uniformly deciding on the use mode (in the presence of receiving-side packet loss, i.e., asymmetric packet loss).

We consider cooperative applications that must periodically decide on the operation mode. Since the consensus problem cannot be deterministically solved in the presence of communication failures, the system is doomed to disagree on the operation mode (in the presence of communication failures that are frequent and severe). We bound the period in which the vehicles can be unaware of such disagreements with respect to the operation mode. We prove that this bound is no more than one communication round (in a vehicular system that deploys a single-hop network of wireless ad hoc communication). We also validate the proposed protocol through NS-3 simulations.

We exemplify how the proposed solution helps to guarantee safety while attaining high performance when vehicles can communicate. We explain how the proposed solution can be used on vehicular platooning to attain high performance and still guarantee high safety standards despite communication failures, such as receiving-side packet loss.

### 1.3. Document Structure

We list our assumptions and define the two problem statements (Section 2, Section 3) before providing the timed protocol for cooperation with disagreement correction (Section 4) and its correctness proof (Section 4). As protocol evaluation study, we exemplify its use in the cooperative vehicular application platooning and validate using computer simulation (Section 5) before the conclusions (Section 6).

## 2. System Settings

A vehicular system that uses a vehicle-to-everything network can be seen as a message passing system that includes a set of *n* participating members that exchange messages with each other. We assume that each vehicle $p_{i}$ has access to the a unique network identifier, *i*. We consider a fully connected network where each vehicle can communicate with all nearby vehicles. We assume the access to a group membership service [15,16,17] that monitors the set of recently live and connected participants as well as the existence of a best-effort (single round) dissemination (gossip) protocol. Moreover, every system component has access to a common global clock by calling the function clock() (such sub-microsecond offsets are widely available and could be implemented, say, using global positioning systems, GPS). We denote by $S_{bound}$ the maximum time difference between the clocks that these vehicles have. For the sake of presentation simplicity, we also assume access to a time yet unreliable dissemination protocol, such as [21,22,23,24,25]. Namely, the algorithm code of vehicle $p_{i}$ in members uses $gossipSend_{i}\left(m\right)$ to broadcast message *m* to all vehicles in the set of members within a known end-to-end delay, *D* (that can be omitted from the network in an arbitrary manner). In other words, the network either delivers messages within *D* from their sending time or omits them in an adversarial manner. We note that different vehicle-to-everything communication systems support bounded *D* values. For example, dedicated short-range communications (DSRC) considers 100 ms scale values. Recent advances in Device-to-Device (D2D) Communication consider 10 ms scale values, where adversarial packet loss can model message delivery delays that exceed these values, say, upon the handover occurrence and during not-in-coverage periods.

We assume that the communication channels and processors are correct. In other words, there are no arbitrary failures neither in the processes nor the communication channel. Therefore, all the received messages are sent by members of the system and arrived without modifications. Further, processors do not alter the memory arbitrarily.

The algorithm code of vehicle $p_{i}$ uses the event $gossipReceive_{i}\left(j,m\right)$ for receiving message *m* from $p_{j}$, see Algorithm 1. We consider synchronous communication rounds of $R_{length}>D+2S_{bound}$.

Every vehicle $p_{i}$ executes a program that is a sequence of *(atomic) steps*. An input event can be either the receipt of a message or a periodic timer going off triggering $p_{i}$ to start a new iteration of the do forever loop.

## 3. The Task

In this section, we present the requirements that the system must satisfy. We start by categorizing the communication periods according to whether vehicles receive all the messages. Next, we present the task requirements.

We define the uncertainty period as the period that vehicles might disagree. We say that there was a communication failure at round *r* if there exists a vehicle that has not received at least one message from any other vehicles during round *r*.

**Definition** **1** (Stable Communication Period). *A stable communication period $X[r_{1},r_{2}]$ is the period of $r_{2}-r_{1}$ rounds in which the vehicles do not experience communication failures, i.e., all vehicles receive all messages during these periods. Otherwise, it is called an unstable communication period, denoted by $Y[r_{1}^{\prime },r_{2}^{\prime }]$.*

**Figure 1:**
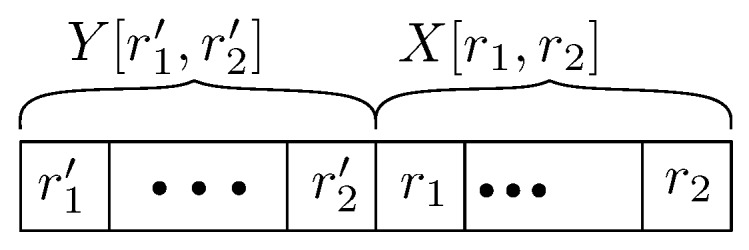
Maximal unstable communication period $Y[r_{1}^{\prime },r_{2}^{\prime }]$ followed by a maximal stable communication period $X[r_{1},r_{2}]$. where $r_{1}=r_{2}^{\prime }+1$

We say that a stable communication period $X[r_{1},r_{2}]$ is maximal if rounds $r_{1}-1$ and $r_{2}+1$ are in unstable communication rounds. Analogously, we define a maximal unstable communication period $Y[r_{1}^{\prime },r_{2}^{\prime }]$; see Figure 1. Thus, in any run, the communication may go infinitely often through a maximal stable period to a maximal unstable period and back to another maximal stable period. The system task is to satisfy requirements 1 to 3.

**Definition** **2** (Requirements).

*1.*  Certainty Period. *Within a bounded prefix of every maximal stable period, there is a suffix which all vehicles operate in cooperative mode.*
*2.*  Bounded Uncertainty Period. *During a maximal unstable period, there may be unstable bounded periods where vehicles operate in different modes.*
*3.*  Bounded Disagreement Correction. *Within a bounded suffix of every maximal stable period, every vehicle operates in autonomous mode.*

Requirement 1 deals with the best cases scenario, i.e., when messages are delivered in a timely manner, all vehicles will perform in cooperative mode. Requirement 2 deals with the worst case scenario, i.e., any period in which vehicles disagree due to failures in the communication is bounded. While requirement 3 deals with the transition from the best case scenario to the worst case scenario.

We show that the execution of Algorithm 1 fulfills requirements 1 to 3. Specifically, by the end of this paper, we demonstrate Theorem 1.

**Theorem** **1.** *Algorithm 1 fulfills requirements 1, 2 and 3, where the uncertainty period is bounded by one round. Moreover, if vehicles do experience communication failures, the disagreement correction holds for at most one round.*

## 4. Timed Protocol for Cooperation with Disagreement Correction

We present the communication protocol that fulfills the requirements. In the protocol, participants exchange messages until a deadline. These messages can include information, for example, about nearby vehicles as well as the confidences that each vehicle has about its information. Once everybody receives the needed information from each other, the participants can locally and deterministically decide on their actions. In the case of a communication failure, each participant that experiences a failure imposes the autonomous operation mode for at least one round.

Each vehicle $p_{i}\in members$ executes the protocol presented in Algorithm 1. The algorithm implements a round base solution. It accesses the global clock and checks whether it is time for the vehicle to send information about the current round (Algorithm 1 , line 15). A vehicle starts sending messages at a $S_{bound}$ time from the beginning of each round and $S_{bound}+D$ before the end of each round using the gossipSend() interface (line 16). Recall that $S_{bound}$ is the maximum time difference over the vehicles and *D* is the longest time that a message can live in the network. Next, it tests whether the current round number *r* points to the current round in time (line 18). A new round starts when clock()÷D is greater than *r*.

At the beginning of every round, the protocol first keeps a copy of the collected data from and the received information in the set data, and updates the round counter, it then nullifies data and sets the operation mode to autonomous (lines 19–21). Then, it tests whether everybody has the same operation mode (line 22). Suppose that there is no communication failure in the previous round and there is not a disagreement. The protocol sets the operation mode to be sent to the cooperative (line 23). It also writes to the pathPlanning() interface the received information as well as the operation mode (line 26).

The proposed protocol interfaces with the gossip (dissemination) protocol by sending messages (gossipSend()) and receiving them (gossipReceive()) periodically. The protocol locally stores the arriving information from $p_{j}\in members$ on each round in data[j] and waits for the round to end before it finishes accumulating all arriving information. More specifically, for each message that is reported with the same round, the protocol stores the data from $p_{k}$, if the message comes directly from $p_{j}$, (k=j), or transitively from $p_{j}$ where (k≠i).

The correctness proof shows that, in the presence of a single communication failure, there could be at most one disagree round in which vehicles operate in different modes. Moreover, the influence of that single failure will last for at most two rounds, which is the shortest period possible. Note that Algorithm 1 handles well any sequence of communication failures.

**Algorithm 1** Timed Protocol for Cooperation with Disagreement Correction (code for $p_{i}$)
1:
**Constants:**
2:$members=\{p_{1},p_{2},\ldots ,p_{n}\}$: the system vehicles.3:⊥: the unavailability symbol denotes a void entry, and the default return value.4:$S_{bound}$: the maximum offset among vehicles.5:*D*: the maximum time that a message time can live in the network.6:$R_{length}>2S_{bound}+D$: round size7:
**Variables:**
8:r←0: Current communication round.9:data[n]={…}: Application data where data[k] is a set that consists of the current operative mode and the state of vehicle *k* at round *r* from member $p_{k}$.10:
**Interface**
11:gossipSend(): Disseminate information to the members.12:gossipReceive(): Dispatch arriving messages.13:readState(): Return the state of the vehicle.14:pathPlanning(): Computes the path according to the operational mode and the state of the system.15:
**Upon** $gossipReceive(j,<r_{j},data_{j}>)$
16:
**if** $\left(r=r_{j}\right)$ **then**
17: **for all** $p_{k}\in members$ **do**18:  **if** ($data_{j}\left[k\right]\neq ⊥$ **and** i≠k) **or** (k=j) **then**19:   $data\left[k\right]\leftarrow data_{j}\left[k\right]$20:  **end if**21: **end for**22:
**end if**
23:
**loop**
24: **if** $clock\left(\right)\in (R_{length}\cdot r+S_{bound},R_{length}\cdot \left(r+1\right)-\left(S_{bound}+D\right))$ **then**25:  gossipSend(i,<r,data>)26: **end if**27: **if** $r<clock\left(\right)÷R_{length}$ **then**28:  $\left(s,r\right)\leftarrow (data,clock\left(\right)÷R_{length})$29:  data←{⊥,…}30:  mode←autonomous31:  **if** $s\left[i\right].mode=s\left[j\right].mode\forall p_{j}\in members$ **then**32:   mode←cooperative33:  **end if**34:  data[i]←{mode,readState()}35:  pathPlanning(s,mode)36: **end if**37:
**end loop**



### 4.1. Correctness

In this section, we show that Theorem 1 is correct. First, we introduce a variant of the consensus problem, called (k,f)-stream consensus, that is suitable for a system that requires to reach consensus infinitely often in a stream of proposed values where the communication channel is unreliable. (k,f)-stream consensus is a synchronous system of n≥2 vehicles where they have to reach at least k−f consensus in *k* consecutive rounds. Intuitively, (k,f)-stream consensus allows having a disagreement in up to *f* rounds among *k* consecutive rounds. Next, we use (k,f)-stream consensus to show the correctness of Theorem 1.

**Problem** **1** ((k,f)-stream consensus). *It is a synchronous system of n≥2 vehicles with the following properties:*

- **Agreement.** *During a period of k rounds, all vehicles must agree on the operation mode for at least k−f consecutive rounds where f<k.*
- **Validity.** *If everyone proposes the cooperative mode during any stable communication round, then the agreed upon value is the cooperative mode.*
- **Termination.** *At the end of each round, every vehicle must decide on the operation mode.*

Observe that (1,0)-stream consensus is equivalent to the classic consensus problem in synchronous systems. Therefore, (1,0)-stream consensus cannot be deterministically solved if the communication channel is unreliable. However, we will show that (2,1)-stream consensus can be deterministically solved. In other words, we show that in any two consecutive rounds, vehicles always reach a consensus in at least one round. Specifically, we show Theorem 2.

### 4.2. Theorem 2 and Its Correctness

**Theorem** **2.** *Algorithm 1 deterministically solves (2,1)-stream consensus.*

Before providing the proof of Theorem 2, we introduce some lemmas. Let $s_{i,j}\left(r\right)$ be the messages that vehicle $p_{i}$ receives from $p_{j}$ at round *r*. Let $s_{i,j}\left(r\right).mode$ be the operation mode of *j* during round *r*. First, we will show that at any round *r*, vehicles only receive messages of the same round from other vehicles.

**Lemma** **1.** *During round r, vehicles do not receive messages from a different round than r.*

**Proof.**  Observe that lines 19–26 are executed when $r<clock\left(\right)÷R_{length}$. However, since *r* is set to $clock\left(\right)÷R_{length}$ in line 19, they are executed only once at the beginning of each round. When a vehicle starts a new round, all the messages received in the previous round are stored in the temporal variable *s* in line 18 before nullfying the set data in line 20. Therefore, the path planning is obtained from the messages received during round r−1.

It remains to show that vehicles at round *r* do not receive messages from a different round. For the sake of contradiction, assume that vehicle $p_{i}$ is at round *r* but it receives a message sent by $p_{j}$ at a different round. Let us first consider the case where $p_{i}$ receives a message that $p_{j}$ sent at round r−1. From the assumptions, $|clock_{i}\left(\right)-clock_{j}\left(\right)|\leq S_{bound}$ and a message takes at most *D* time to be delivered (if it is delivered). Hence, the message must have been sent by $p_{j}$ at time

(1) $$\begin{array}{c} \begin{array}{cc} clock_{j}\left(\right) & \geq clock_{i}\left(\right)-S_{bound}-D\\ & \geq R_{length}\cdot r-\left(S_{bound}+D\right). \end{array} \end{array}$$

From line 15, $p_{j}$ sends a message of round r−1 if

(2) $$\begin{array}{c} clock_{j}\left(\right)<R_{length}\cdot \left(r-1\right)-\left(S_{bound}+D\right). \end{array}$$

By substituting (2) in (1) and factorizing we have a contradiction since r−1≱r. Now assume that $p_{i}$ receives a message that $p_{j}$ sends at round r+1. Therefore, the message must be sent by $p_{j}$ at time

(3) $$\begin{array}{c} \begin{array}{cc} clock_{j}\left(\right) & \leq clock_{i}\left(\right)+S_{bound}\\ & \leq R_{length}\cdot r+S_{bound}. \end{array} \end{array}$$

From line 15, $p_{j}$ sends a message of round r+1 if

(4) $$\begin{array}{c} clock_{j}\left(\right)>R_{length}\cdot \left(r+1\right)+S_{bound}. \end{array}$$

By substituting (4) to (3) and factorizing we have a contradiction since r+1≰r.

 ☐

From the previous lemma, all the messages that $p_{i}$ receives during round *r* are sent during round *r*. We show in the following lemma that Algorithm 1 attains common knowledge during a stable communication period.

**Lemma** **2** (Common knowledge in stable communication period). *Let $X[x_{1},x_{2}]$ be a maximal stable communication period. Then, $s_{i,j}\left(r\right)=s_{k,j}\left(r\right)$ for all $p_{i},p_{j},p_{k}\in members$, where $r\in [x_{1},x_{2}]$.*

**Proof.**  First, we show that data[i] in Algorithm 1 remains consistent on $p_{i}$ during round *r*. Assume for the sake of contradiction that vehicle $p_{i}$ changes its data[i] when receiving a message from vehicle $p_{j}$. It must be that k=i since it loads data[k]. However, since the condition ensures that it loads data[k] only if either i≠k or k=j, it must be that k=j. This is a contradiction since i=j=k and messages are not modified. Therefore, data[i] remains consistent on $p_{i}$ during round *r*.

We say that a message $s_{i,k}\left(r\right)$ is sent transitively if $p_{i}$ receives $s_{i,k}\left(r\right)$ from $p_{j}$ where j≠k. We show that the message transitivity maintains the consistency of the messages during a stable communication period. We argue by contradiction. Assume that $p_{i}$ receives two messages, $s_{i,k}\left(r\right)$ and $s_{i,k}^{\prime }\left(r\right)$ such that $s_{i,k}\left(r\right)$ is sent by $p_{k}$, $s_{i,k}^{\prime }\left(r\right)$ is sent by $p_{j}$ and $s_{i,k}\left(r\right)\neq s_{i,k}^{\prime }\left(r\right)$. Consider the first time that $s_{i,k}\left(r\right)$ and $s_{i,k}^{\prime }\left(r\right)$ were sent. Observe that $p_{k}$ sent the two messages. This is a contradiction since $p_{k}$ maintains consistent its own information over each round and the messages are not modified.

From Lemma 1, at the end of the round *r* the value of data are identical in each vehicle and stored in *s*. Therefore, it holds that $s_{i,j}\left(r\right)=s_{k,j}\left(r\right)$ for all $p_{i},p_{j},p_{k}\in members$. ☐

**Lemma** **3.** *Assume that at a round r due to message lost there are two sets of vehicles where one set is in the autonomous operation mode and the other set is in cooperative operation mode. Then at round r+1, every vehicle agrees on the autonomous operation mode.*

**Proof.**  Let *A* be the subset of vehicles that are in the autonomous operation mode in round *r* and let *C* be the subset of vehicles that are in the cooperative operation mode in round *r*. Observe that each vehicle $p_{i}\in A$ sends the autonomous operation mode and each vehicle $p_{j}\in C$ sends the cooperative operation mode in round *r*. At the beginning of round r+1, each vehicle $p_{i}$ sets the operation mode according to the following rule:

$$mode_{i}\left(r+1\right)=\left\{\begin{array}{cc} cooperative & \;\mathrm{if}\;s_{i,i}\left(r\right).mode=s_{i,j}\left(r\right).mode\\ & \;\;\;\forall j\in members\\ autonomous & \;\mathrm{otherwise} \end{array}\right.$$

Consider $p_{i}\in A$. $p_{i}$ either receives the cooperative mode from $p_{j}\in C$ or it does not receive it. In both cases, $p_{i}$ decides autonomous mode since $s_{i,i}\left(r\right).mode\neq s_{i,j}\left(r\right).mode$. Now consider $p_{j}\in C$. $p_{j}$ either receives autonomous from $p_{i}$ or it does not receive the message. In both cases $p_{i}$ decides autonomous mode since $s_{j,j}\left(r\right).mode\neq s_{j,i}\left(r\right).mode$.

 ☐

We are ready to prove Theorem 2.

**Proof.** (**Theorem 2**)

1. **Agreement.** We have to prove that in any two consecutive rounds r,r+1, vehicles can disagree on the operation mode in at most one communication round. Observe that if they agree on the operation mode in round *r*, then the property holds. Assume then that vehicles disagree on the operation mode in round *r*. However, from Lemma 3, vehicles agree on the operation mode in round r+1.
2. **Validity.** It follows directly from Lemma 2.
3. **Termination.** It easily holds since at the beginning of each round, all vehicles decide the operation mode (lines 18–26).

 ☐

### 4.3. Correctness of Theorem 1

We show in Theorem 2 that in every two consecutive rounds, the active vehicles deterministically agree on the operation mode of at least one round in every two consecutive rounds even if they cannot communicate. We now use Theorem 2 to prove the main result of the paper.

**Proof.** (**Theorem 1**)

First we show that Algorithm 1 fulfills Requirement 1 of Definition 2 (Certainty Period). Consider every maximal stable communication period $\left[r_{1},r_{2}\right]$. From Lemma 2, vehicles have common knowledge, i.e., $s_{i,j}\left(r\right)=s_{k,j}\left(r\right)$ for all $p_{i},p_{j},p_{k}\in members$ where $r\in [r_{1},r_{2}]$. Therefore, during $\left[r_{1}+1,r_{2}+1\right]$ all vehicles decide to operate in cooperative mode.

Observe that the disagreement correction holds for the first round in the maximal stable communication period, i.e., $r_{1}$. Therefore, Algorithm 1 fulfills property 3 (Bounded Disagreement Correction).

To show that Algorithm 1 fulfills property 2 (Bounded uncertainty round) consider a maximal unstable period $Y[r_{2}+1,r_{3}]$. From Theorem 2 Algorithm 1 deterministically solves (2,1)-stream consensus in the period $Y[r_{2}+1,r_{3}]$. Therefore, vehicles may operate in different modes for at most one consecutive round. The theorem follows. ☐

## 5. Evaluation

We exemplify the use of Algorithm 1 in the vehicular application platooning also known as cooperative advance cruise control. In platooning, vehicles keep short headways (the distance between two cars expressed as time) to reduce the air resistance. Since the gas consumption is directly proportional to the air resistance, it is desirable to minimize the headways. However, keeping short headways can be risky if vehicles behave totally autonomously. For example, rear-end crash events as well as near-crash events usually involve an action of the lead vehicle [26]. Vehicles can reduce the headway without risk by sharing their vital information such as acceleration. To see this, assume a vehicular system with *n* vehicles where each vehicle has a reaction time of at least *T* units of time. Consider the case where vehicles are autonomous. During an emergency brake of the leader, the last vehicle requires a headway of at least Ω((n−1)T). To see this, observe that the second vehicle reacts after Ω(T) time, the third vehicle reacts after Ω(T) time of the second vehicle, i.e., Ω(2T). Generalizing, the vehicle *i* reacts after time Ω((i−1)T). Consider now the case of cooperative vehicles. Observe that the reaction time is the time that all nearby vehicles require to agree, say $T^{\prime }$. Therefore, the last vehicle requires a headway of at least $\Omega (T^{\prime })$. Observe that the cooperative system is scalable while the autonomous system is not. However, due to communication failures, vehicles may have inconsistent information.

We exemplify how Algorithm 1 can be used to implement a cooperative vehicles application that can deal with communication failures. Our design demonstrates that, even though the presence of communication failures can lead to disagreement about what mode vehicles should operate in for a particular communication round, this can only happen for a period of at most one round, and thus is tolerated by the vehicular control algorithm.

We do not aim at designing new vehicular systems, but rather to exemplify how the proposed solution helps to guarantee the safety in existing cooperative vehicular applications, which operate in environments that include communication uncertainties. In our approach, while the vehicles are aware that nearby vehicles have a high level of certainty, they perform a fully cooperative operational mode to improve their performance. However, when this cannot be determined beyond any doubt, they switch to the autonomous operational mode to maintain high safety levels.

In platooning, the vehicles exchange their vital information which contains the vehicles’ velocity vector, sensor errors as well as operational parameters. The errors are used to determine the headway that vehicles should keep.

Note that even though our example considers a cooperative operation mode with two distinct headways, the extension to a scheme with more distinct headways is straightforward.

Algorithm 2 implements the interface functions readState and pathPlanning of Algorithm 1. The function readState returns the $p_{i}$’s local state meanwhile the main functionality is implemented in the pathPlanning function. At the beginning of each round, Algorithm 1 calls readState in Algorithm 2 to obtain the information that the cooperative vehicle platooning requires to operate. At the end of the round, Algorithm 1 reaches a decision of the operation mode to operate in the following round independently of the errors and calls pathPlanning of Algorithm 1 which acts accordingly.

We assume the existence of safe algorithms for each operation mode provided that the information meets the requirements, i.e., the errors are within the bounds that are given in Table 1.

**Algorithm 2** Cooperative vehicle platooning with ACC as a base-line application (code for vehicle $p_{i}\in members$).
1:**Executes** the protocol that Algorithm 1 presents.2:
**function** readState()
3:Let $S=\{pos,speed,pos_{error},speed_{error}\}$ be the position, speed, position error and speed error.4:
**return** *S*
5:
**function** pathPlanning(data,mode)
6:**if**$p_{i}$ is the platoon leader **then**7: follow the original path8:
**else**
9: Let $posMax_{error}=\max_{\forall p_{j}}\left(data\left[j\right].pos_{error}\right)$10: Let $speedMax_{error}=\max_{\forall p_{j}}\left(data\left[j\right].speed_{error}\right)$11: **if** mode=COOPERATIVE **and** $posMax_{error}\leq L$ **and** $speedMax_{error}\leq S$ **then**12:  Switch to the cooperative mode and use speed and position to keep headway $H_{1}$13: **else if** mode=COOPERATIVE **and** $posMax_{error}\leq L$ **and** $speedMax_{error}\geq S$ **then**14:  Switch to the cooperative mode and use speed and position to keep headway $H_{2}$15: **else**16:  Switch to the autonomous mode and keep headway $H_{3}$17: **end if**18:
**end if**



The safety provision in Algorithm 2 depends directly on the mechanical constraints and the parameters’ election. Observe that during the cooperative operation mode, every vehicle keeps the same headway since they have common knowledge.

From the previous section, it is reasonable to consider rounds of length at least 260 ms . Thus, the headway can be determined from the round length and the error bounds on the information.

### Simulation

In this subsection, we simulate the protocol in NS-3 (http://www.nsnam.org/) to verify Algorithm 1. First, we simulate under frequent communication failures to verify that the vehicles disagree for no more than one consecutive round. Next, we simulate for three different round lengths and various vehicles to obtain the round length that allows the vehicles to operate in cooperative mode the majority of the time.

We consider a vehicular system where vehicles can operate in either cooperate or autonomous mode. The autonomous operation mode is the default mode to which the system falls-back to in the presence of communication failures. We implement the algorithm to validate the protocol as well as its efficiency. For the efficiency, we consider the *reliability* measure, which we define as the percentage of communication rounds during which the protocol allows the system to operate in the cooperative mode. First, we validate that the disagreement period consists of at most one round and we move next to the reliability of the protocol.

Observe that when the round length is long enough to exchange several messages, it is more probable that the system operates in cooperative mode. Meanwhile, when the round length is short, it is less probable. However, longer round length implies longer disagreement period. Thus, there is a trade-off between the disagreement period and the round length. Therefore, a natural question is to determine a round length that provides a good trade-off. To measure the efficiency, we consider the *reliability* which we define as the percentage of communication rounds during which the protocol allows the system to operate in the cooperative mode given a round length. To determine the round length, we implement Algorithm 1 where vehicles can operate in either cooperative or autonomous mode.

We simulate Algorithm 1 using NS-3 (http://www.nsnam.org/). We choose IEEE 802.11p as the communication channel with a log-distance path loss model and Nakagami fading channel model. Since DSRC technologies support end-to-end message delay of less than 100 ms [27], we fix the message delay to 100 ms. We consider a synchrony bound of 5 ms, say, using GPS [28] or a distributed clock-synchronization protocol. We implement a straightforward gossip protocol in which every node retransmits the message every 50 ms.

First we validate that the disagreement period is of at most one round with 4 vehicles. We set the round length to 160 ms so that messages can be transmitted twice in each round. Recall that the round length is at least the message delay (100 ms) plus twice the synchrony bound (5 ms). Since the round length allows only one retransmission, the system experiences frequent communication failures.

**Figure 2:**
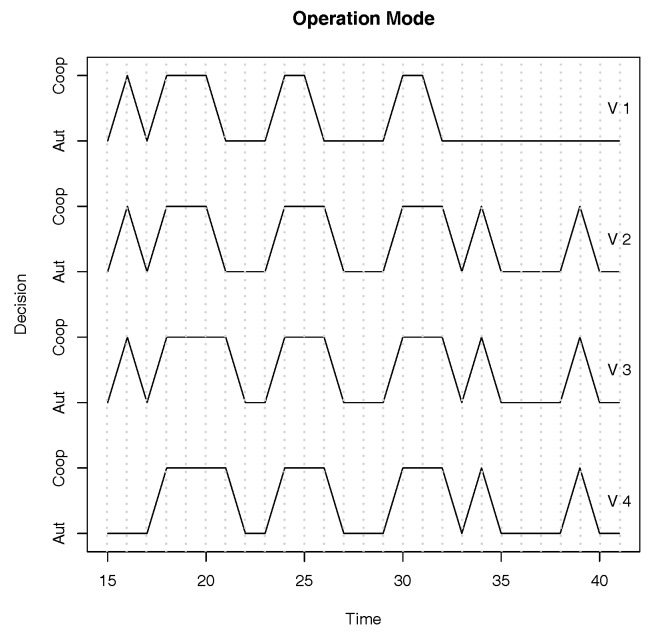
Vehicle behavior under frequent communication failures. The plot shows the decision that four vehicles took among two operation modes during 25 rounds using Algorithm 1.

We plot in Figure 2 the decision that the 4 vehicles took independently during 25 rounds using the protocol under frequent communication failures. Observe that at round 21 vehicles 1 and 2 change to autonomous operation mode due to a communication failure, but vehicles 3 and 4 still continue in the cooperative operation mode. However, at round 22, they change to the autonomous operation mode. Although vehicles do not operate on distinct modes for more than one round, the operation mode of some vehicles may be oscillating. We can reduce this effect by increasing the round length. However, the uncertainty period also increases.

Note the trade-off between the upper bound on the disagreement period, which is one communication round, and the success rate of the gossip protocol, which decreases as the round length becomes shorter. The type of gossip protocol as well as the number of system components also influences this success rate. We use computer simulation to study how these trade-offs work together and present the reliability.

To determine a round length, we execute the simulation with rounds of length 160,260 and 360 so that the involved vehicles can transmit 2,4 and 6 messages in each round, respectively. Further, we variate the number of vehicles between two and eight to verify the effects on the proposed protocol. We run each experiment for 360 simulation seconds.

**Figure 3:**
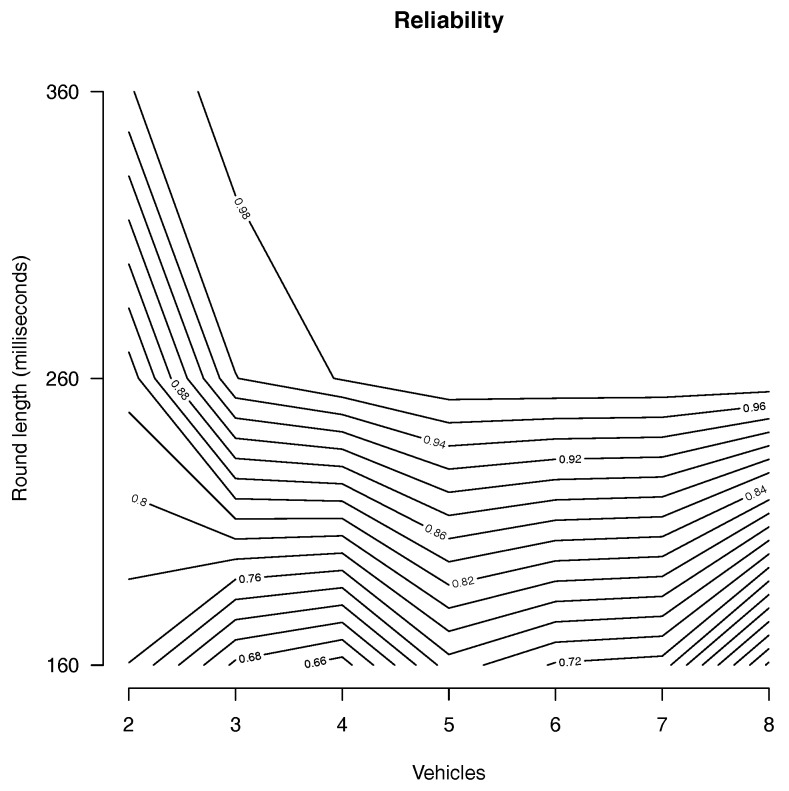
The percentage of time that all vehicles agree on the cooperative operation mode (number of vehicles vs. the round length in milliseconds).

During the simulations, we observe an average packet drop of 0.1436347. The reliability is plotted in Figure 3 where the *x* axis represents the number of vehicles, the *y* axes represents the round length and the contour lines represent the reliability. We can observe from the plotting that for round length of 260 ms, the vehicles operate in cooperative mode for 98% of the time when more that 3 vehicles are involved. However, it is reduced to 94% and 82% with three and two vehicles, respectively. Observe that the reliability increases with more vehicles. For instance, with 4 vehicles, Algorithm 1 maintains the cooperative operation mode 98% of the time. Meanwhile with 2 vehicles, the cooperative operation mode is reduced to 82%. This is because of the transitivity property of the gossip protocol. Nevertheless, during the percentage of time that Algorithm 1 cannot maintain the cooperative operation, the safety of the system is kept by guaranteeing that the vehicles do not disagree for more than one consecutive round.

For the platooning vehicular application previously presented, it is reasonable to have rounds of length at least 260 ms with headway of at least 520 ms.

## 6. Conclusions

We have proposed an efficient protocol that can be used in safety-critical cooperative vehicular applications that have to deal with communication uncertainties. The protocol guarantees that all vehicles will not be exposed, for more than a constant time, to risks that are due to communication failures. We demonstrate correctness, evaluate performance and validate our results via NS-3 simulations.

The proposed solution can be applied to implementing cooperative vehicular applications, such as intersection crossing, coordinated lane change, platooning, as we demonstrated using the Gulliver test-bed [29] during the KARYON project [30]. (Demonstration videos are available via http://www.chalmers.se/hosted/gulliver-en/documents.) Moreover, we have considered the simplest multi-hop communication primitive, i.e., gossip with constant retransmissions. However, that communication primitive can be substituted with a gossip protocol that facilitates a greater degree of fault-tolerance and better performance. This work opens the door for the algorithmic design and safety analysis of many cooperative applications that use different high-level communication primitives.

## Figures and Tables

**Table 1:** *L*, *S* and $H_{\left(\cdot \right)}$ are constant values known by all participants such that $H_{1}<H_{2}<H_{3}$.

Headway	Localization Error (${Pos}_{\epsilon }$)	Speed Error (${Speed}_{\epsilon }$)
$H_{1}$	$Pos_{\epsilon }\leq L$	$Speed_{\epsilon }\leq S$
$H_{2}$	Unbounded $Pos_{\epsilon }$	Unbounded $Speed_{\epsilon }$
$H_{3}$	Unbounded $Pos_{\epsilon }$	Unbounded $Speed_{\epsilon }$
